# Growth of Yellowtail (*Seriola quinqueradiata*) Fed on a Diet Including Partially or Completely Defatted Black Soldier Fly (*Hermetia illucens*) Larvae Meal

**DOI:** 10.3390/insects12080722

**Published:** 2021-08-12

**Authors:** Atsushi Ido, Muhammad-Fariz-Zahir Ali, Takayuki Takahashi, Chiemi Miura, Takeshi Miura

**Affiliations:** 1 Graduate School of Agriculture, Ehime University, Matsuyama 790-8566, Japan; ido@agr.ehime-u.ac.jp (A.I.); fariz290392@gmail.com (M.-F.-Z.A.); takataka@dpc.ehime-u.ac.jp (T.T.); c.miura.6u@it-hiroshima.ac.jp (C.M.); 2 Department of Global Environment Studies, Faculty of Environmental Studies, Hiroshima Institute of Technology, Saeki 731-5193, Japan

**Keywords:** yellowtail, black soldier fly, defatting, fish meal replacement, lauric acid, fat fraction

## Abstract

**Simple Summary:**

Insects which can be reared artificially, such as the black soldier fly, housefly or yellow mealworm are considered as promising feed sources for sustainable aquaculture. The present study is the first to reveal the potential of diets containing insect meal for juvenile yellowtail. The growth of fish fed diets in which fish meal was replaced by 10–30% partially defatted black soldier fly larvae meal was decreased in accordance with the content of the larvae meal. On the other hand, growth of fish with a diet including 20% completely defatted larvae meal was equivalent to that with a diet of the partially defatted larvae meal. Thus, the fat fraction of black solider fly larvae could cause growth retardation of yellowtail, and the defatting process of the insect meal may be important in the manufacture of black soldier fly larvae meal for yellowtail.

**Abstract:**

Against a background of increased demand for fish meal (FM), black soldier fly larva is a promising alternative feed source for sustainable aquaculture. Yellowtail, the most popular farmed fish in Japan, is a carnivorous fish; therefore, it requires a high proportion of FM in its diet. This study represents the first example of yellowtail fed on a diet including insect meal as a replacement for FM. Partially defatted black soldier fly meal (PDBM) comprised 49.0% crude protein and 23.2% crude fat, while completely defatted black soldier fly meal (CDBM) contained less than 10% crude fat, as the same level as FM was achieved with defatting PDBM using hexane. In feeding trials, growth of the fish was reduced in accordance with PDBM content: 10%, 20%, and 30% in their diet. Although a diet including 8% CDBM (with the same protein composition as 10% PDBM) also resulted in decreased fish growth, growth with a diet including 16% CDBM (with the same protein composition as 20% PDBM) was significantly higher than that of 20% PDBM, and equivalent to that of 10% PDBM. Therefore, even 10% of partially or completely black soldier fly larvae meal in diets inhibited growth in juvenile yellowtail, and we found that removal of the fat fraction could improve fish growth.

## 1. Introduction

Fish meal (FM) is an essential component in aquaculture feed. The use of the aquaculture feed is estimated to keep increasing until 2025 due to a rapid growth in world aquaculture production, and the price of FM has been steadily increasing [1]. About 30 million tons of wild-caught pelagic fish per year were used for FM production, and pressure on the fish from fishing is a serious concern for marine ecosystems [2]. Thus, a development of an alternative feed source for cultured fish is an urgent issue.

Insects are thought to be a potential feed source for sustainable aquaculture [3,4,5]. Black soldier fly (BSF) (*Hermetia illucens*) larvae are one of the most promising insect species used for fish feed. BSF larvae can be artificially reared in a highly efficient way using organic by-products, and they contain a high proportion of protein [3]. BSF larvae offer more than just a protein source. A novel bioactive polysaccharide that can stimulate innate immunity was identified in BSF larvae [6], while dietary BSF larvae were reported to provide improvements in immunity and disease resistance in barramundi (*Lates calcarifer*) [7] and European seabass (*Dicentrarchus labrax*) [8].

Aside from freshwater species, however, the replacement of FM with BSF larvae meal has not always been successful in carnivorous marine fish species. The complete replacement of 10% of FM with 14.8% of BSF larvae meal resulted in no difference in growth in Atlantic salmon (*Salmo salar*) [9,10], while the replacement of dietary FM with more than 16.5% BSF larvae meal reduced the growth of juvenile turbot (*Psetta maxima*) [11]. A dietary inclusion level of BSF larvae meal that does not adversely affect Japanese seabass (*Lateolabrax japonicus*) and European seabass is just 19.2% and 19.5% of their diet, respectively [12,13,14]. In these studies, the factors of BSF larvae meal that affected marine fish growth remained unknown.

The aquaculture of fish belonging to the genus *Seriola* is becoming important worldwide [15], with yellowtail (*Seriola quinqueradiata*) in particular the most popular and important cultured fish in Japan [16]. The problem in yellowtail farming is that their diets rely on a high proportion of FM, and little progress has been made in reducing the amount of FM included, and commercial pelleted diet for yellowtail still contains 50–60% FM [17,18,19]. Plant protein, such as soybean meal, has been tried but was found to suppress the secretion of digestive enzymes in yellowtail [20,21]. Therefore, novel animal protein sources that are highly palatable to yellowtail are urgently needed. The replacement of FM with insect meal has been investigated for many cultured fish species; however, the potential of insect meal in feed for yellowtail has not yet been demonstrated.

This study is the first report of juvenile yellowtail being fed diets including insect meal as a replacement for FM. In the study, the effects of partially defatted and completely defatted BSF larvae meals on the growth of yellowtail body have been assessed.

## 2. Materials and Methods

### 2.1. Ethics Statement

Animal experiments were carried out following the guidelines of Ehime University. The study protocol was approved by the Institutional Animal Care and Use Committee (IACUC) of Ehime University (Permit Number: 3908).

### 2.2. Defatting of Black Soldier Fly Larvae Meal

Partially defatted BSF larvae meal (PDBM) produced in Malaysia was kindly provided by Shintoa Corporation (Tokyo, Japan). The defatting of BSF larvae was conducted using press. Briefly, BSF larvae reared with agricultural by-products were washed, dried, ground, and pressed for defatting. To remove the fat fraction from PDBM, we defatted PDBM with hexane by the method that we previously described [22,23]. Briefly, PDBM was suspended with a 4-fold greater volume of hexane, and then incubated overnight at room temperature with occasional gentle agitation. The supernatant was removed with filtration, then the residue was heated at 60 °C for 1 day or more until dry and completely defatted BSF larvae meal (CDBM) was obtained.

### 2.3. Feed Formulation of Experimental Diets

We devised feed formulations based on results of proximate, amino acid, and fatty acid analyses (Table 1 and Table 2). In this study, the diets were formulated not only to be isonitrogenous (46–48% crude protein), but also to adjust fatty acid composition between the experimental diets.

In feeding trial 1, diets including 64% FM were used for two control groups, and three test diets were formulated to contain 10% PDBM (10PD), 20% PDBM (20PD), and 30% PDBM (30PD), replacing 11%, 22%, and 33% of FM in the control diets, respectively. As the fat fraction of PDBM included 29.2% lauric acid in the total fatty acids, palm kernel oil, which is also rich in lauric acid, was added to Control+L, 10PD, 20PD, and 30PD except for the Control to be an equivalent level to lauric acid, while corn oil was contained in the Control diet to equalize the total fat content. Furthermore, the fish oil content was increased in diets including PDBM in consideration of the fat fraction included in FM to be an equivalent level of fat from fish in each diet (Table 1).

In feeding trial 2, test diets including 10% PDBM (10PD) and 10% CDBM (10CD) were formulated to replace 11% of FM, and 20% PDBM (20PD) and 20% CDBM (20CD) were formulated to replace 22% of FM in the control diets, respectively. Palm kernel oil was added to Control+L, 10PD, 10CD, 20PD, and 20CD except for the Control to be an equivalent level to C12:0, while corn oil was included in the Control diet to equalize the total fat content. The fish oil content was increased in diets including PDBM or CDBM to make fat from fish in each diet equal. In other words, all diets contained equivalent levels of fish-derived fat, and a diet of Control+L and test diets including PDBM or CDBM contained equivalent levels of lauric acid (Table 2).

### 2.4. Feeding Trials

Juvenile yellowtails (average body weight: 0.7 g) were kindly provided by Yamasaki Giken Co. Ltd. (Kochi, Japan). The fish were fed a commercial diet (Marubeni Nisshin Feed Co., Ltd., Tokyo, Japan) for about 1 month for acclimation. The ingredients of the experimental diets were well mixed after adding water, granulated to 1.6–3.0 mm diameter, and then air-dried at 60–70 °C for more than 1 day.

For feeding trial 1, at the beginning of the trial, 30 fish (average fork length: 6.6 cm; average body weight: 2.9 g) were distributed to each of ten 200 L tanks that used a flow-through system. Two tanks per group were set. The fish were fed to satiation twice a day, 6 days per week. The fish were cultivated for 84 days (12 weeks). The water temperature varied between 18.8–24.8 °C during the trial. The fork lengths (FL) and body weights (BW) were measured four times (at the start, after 3 weeks, after 6 weeks, and after 12 weeks) under anesthesia with 2-phenoxy ethanol (Nacalai Tesque, Kyoto, Japan). Then, 28 fish per group were randomly selected and their intestinal lengths were measured.

In feeding trial 2, at the beginning of the trial, 30 fish (average fork length: 7.7 cm; average body weight: 5.3 g) were distributed to each of twelve 200 L tanks that used a flow-through system. Two tanks per group were set. The fish were fed to satiation twice a day, and 6 days per week. The fish were cultivated for 35 days (5 weeks). The water temperature varied between 18.9–22.6 °C during the trial. FL and BW were measured three times (at the start, after 2.5 weeks and after 5 weeks) under anesthesia with 2-phenoxy ethanol (Nacalai Tesque, Kyoto, Japan).

FL gain, BW gain, FL gain rate, BW gain rate, specific growth rate (SGR), and feed conversion ratio (FCR) were calculated as follows:

FL gain rate (%) = (FL at measurement − initial FL)/initial FL × 100,

BW gain rate (%) = (BW at measurement − initial BW)/initial BW × 100,

SGR, % day^−1^ = [(ln final BW − ln initial BW)/number of feeding days] × 100,

FCR = total feed intake/(final BW − initial BW)

Measurements from individual fishes were used to obtain the FL, BW, FL gain, BW gain, FL gain rate, and BW gain rate in the feeding test groups, and values in the duplicate tanks were used to obtain the total feed intake per fish and FCR in each study group. Intestinal length ratio (%) was calculated by intestinal length/final FL × 100.

### 2.5. Proximate Composition, Amino Acid, and Fatty Acid Analysis

The proximate composition, amino acids, and fatty acids of the experimental diets or their ingredients were analyzed with the Association of Analytical Communities (AOAC) methods [24]. Briefly, the content of crude protein was analyzed with the Kjeldahl method [24]. “Kjeltab” (containing K_2_SO_4_) was added to the samples, and the samples were digested in a block heater (Tecator^TM^ Digestion Systems 2520, FOSS). The nitrogen content was analyzed using an auto analyzer (Kjeltec^TM^ 8400, FOSS). The crude protein was obtained based on the calculation from the nitrogen content with the nitrogen–protein conversion factor, 6.25. Crude fat was analyzed with the Soxhlet extraction method [24]. Extraction from the samples was conducted with petroleum ether in an Automated extractor (Soxtec^TM^ 8000, FOSS). The content of ash was analyzed with an electric furnace (MMF-1, AS ONE).

Proteinogenic amino acid composition in the samples was analyzed with an automated amino acid analyzer (Shimadzu, Kyoto, Japan) after hydrochloric hydrolysis with sodium chloride. For methionine and cystine, the samples were oxidized with performic acid prior to hydrochloric hydrolysis. For tryptophan, samples were prepared with barium hydroxide octahydrate and thiodiethylene glycol prior to hydrolysis with sodium chloride, then analyzed using high-performance liquid chromatography. Fatty acid composition in samples were prepared with saponification and analyzed using gas chromatography with a method described in food labeling standards produced by the Consumer Affairs Agency Japan (CAA, 2015). The analysis of proteinogenic amino acids and fatty acids was conducted by the Japan Food Research Laboratories (Osaka, Japan).

### 2.6. Statistical Analysis

For FL, BW, FL gain rate, BW gain rate, and intestinal length ratio on the feeding trial 1 and 2, statistically significant differences among the control and test groups were identified by the Kruskal–Wallis test and Mann–Whitney test with Holm’s correction for post hoc comparison (*p* < 0.05). The tests were conducted using R software (https://www.r-project.org (accessed on 10 May 2021)).

## 3. Results

### 3.1. Analysis of Proximate Composition, Amino Acid Profiles, and Fatty Acid Profiles

The proximate compositions of BSF larvae meals used in this study are shown in Table 3. PDBM contained 49.0% crude protein (CP) and 23.2% crude fat (CF). CDBM obtained from PDBM defatted with hexane contained 8.3% CF, which was comparable with that present in FM. Although PDBM covered essential amino acids required for fish growth and survival (Table 4), there was a big difference between oil from PDBM and fish oil in terms of their fatty acid profiles. Compared with fish oil, oil from PDBM was rich in saturated fatty acids, especially lauric acid (C12:0), and lacking in polyunsaturated fatty acids (Table 5).

### 3.2. Feeding Trials with Juvenile Yellowtail

**Figure 1:**
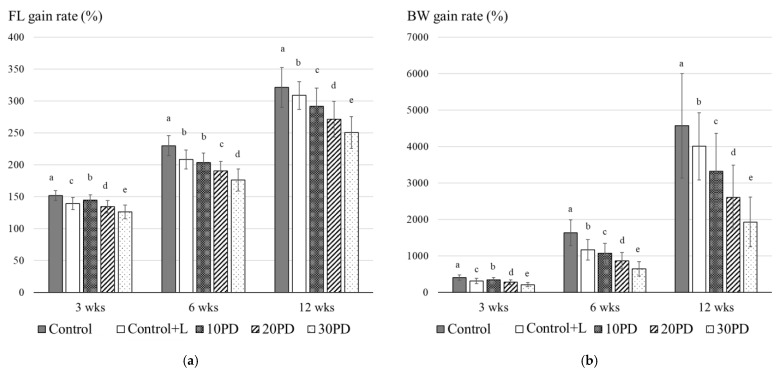
Fork length gain rate (**a**) and body weight gain rate (**b**) of yellowtail in feeding trial 1. Bars indicated S.D. Different letters indicate statistically significant differences according to Kruskal–Wallis test and Mann–Whitney test with Holm’s correction for post hoc comparison (*p* < 0.05). Abbreviations: FL, fork length; BW, body weight.

**Figure 2:**
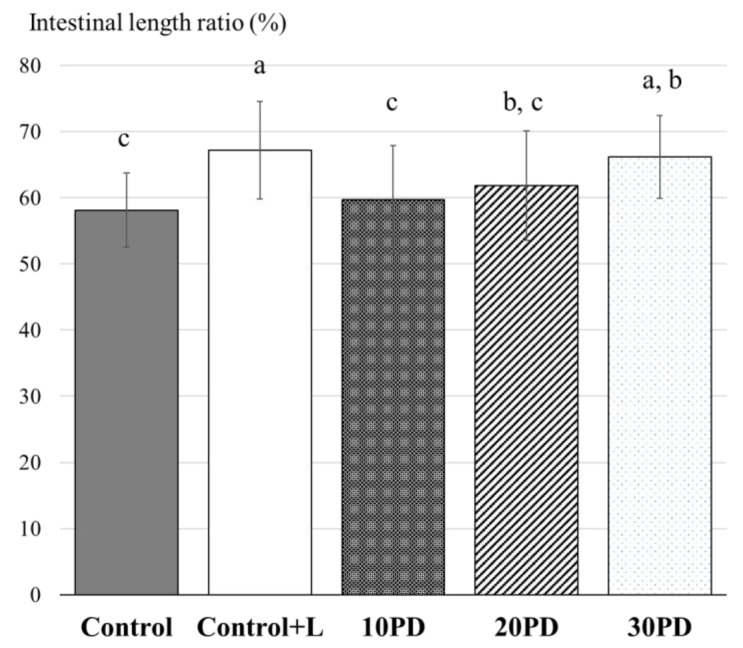
Intestinal length ratio of yellowtail in feeding trial 1. Bars indicated S.D. Different letters indicate statistically significant differences according to Kruskal–Wallis test and Mann–Whitney test with Holm’s correction for post hoc comparison (*p* < 0.05).

Two feeding trials with juvenile yellowtail were conducted. In the first trial, feeds including 10%, 20%, and 30% PDBM were evaluated. In consideration of lauric acid included in BSF larvae meal, we set two control diets; the Control was free from PDBM and lauric acid and the Control+L contained lauric acid at the same level as 10PD, 20PD, and 30PD. The DHA (C22:6) content was equal in all diets (Table 4). Although all diets were isonitrogenous and isolipidic, the growth of fish fed Control+L containing lauric acid was significantly inferior to that of the Control, and the growth decreased with PDBM content. FCR slightly increased in fish fed diets that included PDBM (Table 6; Figure 1). The intestinal length ratio at 12 weeks in each group is shown in Figure 2. The ratios in the Control+L, 20PD, and 30PD groups were significantly higher than that of the Control. Compared with fish fed on the Control diet, the ratio tended to increase with PDBM content.

**Figure 3:**
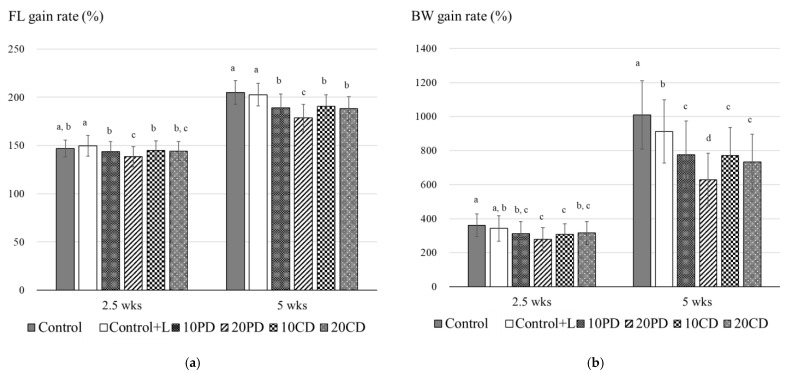
Fork length gain rate (**a**) and body weight gain rate (**b**) of yellowtail in feeding trial 2. Bars indicated S.D. Different letters indicate statistically significant differences according to Kruskal–Wallis test and Mann–Whitney test with Holm’s correction for post hoc comparison (*p* < 0.05). Abbreviations: FL, fork length; BW, body weight.

In the next trial, diets containing CDBM were compared with diets containing PDBM. Growths in fish fed on diets containing PDBM and CDBM decreased significantly comparing with growth in the Control and Control+L groups, and fish growth declined according to the amount of PDBM. However, growth in fish fed on diets including 20% CDBM was similar to that in groups fed on 10% CDBM and PDBM, and significantly higher than that in the group fed on 20% PDBM (Table 7; Figure 3). There was also a significant difference between the two Control diets in this trial, Control+L containing lauric acid in BW gain rate (Figure 3). FCR was almost the same in all groups.

## 4. Discussion

In this study, we demonstrated the feeding trials to explore the potential of BSF larvae meal for yellowtail aquaculture; however, the results showed adverse effects on growth with a diet of even 10% BSF larvae meal content and 11.1% FM substitution. Although other studies of different fish species have concluded that BSF larvae meal could replace FM, the levels of BSF larvae meal included in the diets investigated were limited. Diets with around 10% full-fat BSF larvae included resulted in no adverse effects in Nile tilapia (*Oreochromis niloticus*) [25], while those with partially defatted BSF larvae resulted in the same or higher growth in African catfish (*Clarias gariepinus*) [26], Jian carp (*Cyprinus carpio*) [27], and rice field eel (*Monopterus albus*) [28]. On the other hand, several studies investigated diets with a high content of BSF larvae. Using 29% full-fat BSF larvae meal could completely replace FM for climbing perch (*Anabas testudineus*) [29], while a diet with more than 30% defatted BSF larvae meal negatively affected yellow catfish (*Pelteobagrus fulvidraco*) [30] and Siberian sturgeon (*Acipenser baerii*) [31]. Although a diet that included 40% defatted BSF larvae meal and 50% FM substitution did not affect the growth of rainbow trout (*Oncorhynchus mykiss*) [32], that with 21% full-fat BSF larvae inclusion resulted in adverse effects [33]. In a meta-analysis of 16 peer-reviewed journal publications about BSF, diets that included levels of more than 29% (± 3%) BSF larvae depressed growth performance in fish [34]. Therefore, it is known that growth tends to be reduced in fish fed on diets containing high levels of BSF. However, compared with these previous studies, it should be noted that growth retardation in yellowtail occurred with just 10% BSF larvae meal inclusion and 11.1% FM substitution.

Chitin, a linear homopolymer of β(1-4)-linked N-acetylglucosamine units and a major constituent of insect cuticles, is thought to result in growth reduction in marine fish because it is difficult to digest [11]. Of the total nitrogen in BSF prepupae, 9.4% is reported to be derived from chitin [35]. As the nitrogen in the PDBM used in this study was 7.8%, the amounts of chitin and protein were estimated to be approximately 10.7% and 41.4%, respectively. The total amino acids in the PDBM comprised 40.5% on a dried basis, so this estimate seems to be reasonable. However, the BSF larvae meal in the test diets was only 10–20%, so the content of chitin derived from BSF in the test diets was only between 1–2%. A diet supplemented with 10% chitin does not inhibit growth, and chitinase enzyme activity has been detected in the stomach of yellowtails [36]. Therefore, chitin was not thought to be a major factor responsible for causing the significant reduction in growth.

One factor derived from BSF larvae that can affect fish growth seems to be the fatty acid profile of the diet. Crude fats of BSF larvae and pre-pupae in diets are known to significantly alter gut microbiota in rainbow trout [37]. The BSF larvae meal used in the present study was rich in lauric acid and it comprised 30% of total fatty acids. Lauric acid has a high melting point and is solid at room temperature. Diets that are rich in lauric acid do not affect growth in freshwater rainbow trout [38,39] or Atlantic salmon [40]. In addition, as long as the fatty acid composition in the diet meets the essential nutrition requirements, the diet including lauric acid at 14% of total fatty acid promotes the growth of the yellow croaker (*Larimichthys crocea*) [41]. In contrast, our findings showed that dietary intake of lauric acid could negatively affect the growth of yellowtails. However, the growth in fish fed on diets containing BSF larvae meal was significantly decreased than that in fish fed on the Control+L diet including lauric acid. These results indicate that factors other than fatty acids affect growth. In feeding trial 1, we found that intestinal length ratios in fish fed on diets that included lauric acid were higher than those in the Control, and the length ratios increased with increasing PDBM content. Enlargements in the intestine of fish fed on BSF larvae have also been reported in Atlantic salmon [9], so the BSF larvae meal content or difference in the fatty acid profile of a diet might induce morphological changes in the intestines of fish. No difference in the intestinal length was observed during feeding trial 2 (data not shown). This might be due to the difference in the durations of the feeding period.

The effects of fat fractions other than fatty acids from BSF larvae on fish growth should be concerned. We observed that the growth of fish in the 20CD group was significantly higher than that of the 20PD group, and that it was comparable with that of the 10PD group. Thus, it seems as though the fat fraction of BSF larvae would depress fish growth. In our previous studies with red seabream (*Pagrus major*), yellow mealworm (*Tenebrio molitor*) and housefly (*Musca domestica)* larvae defatted with hexane could successfully replace FM at proportions of 65% and 70%, respectively, in the diet [22,23]. Additionally, dietary intake of catechol identified in fat fraction from housefly larvae causes a reduction in growth and morphological alterations in the intestines in fish [23]. In these studies, as in the present study, fat fractions of insects could possibly depress fish growth. As the diets including CDBM were inferior to the control diets, it is not only the fat fraction but also other factors in BSF larvae that must depress fish growth. As the fat fraction of BSF larvae showed an enhancement of innate immunity, allowing the use of elevated levels of plant proteins in the diet of rainbow trout [42], there is no doubt that differences in the sensitivity among fish species lead to different results.

## 5. Conclusions

We conducted the feeding trials with juvenile yellowtail for the purpose of replacing FM with BSF larvae meal. The fish growth was significantly reduced in accordance with content of BSF larvae meal. However, the growth of fish fed on 20% CDBM was comparable with those on 10% PDBM, so we found that removal of fat fraction from the meal could improve the fish growth. Regardless of finding negative effects on yellowtail growth in this study, insects are still considered to be useful feed ingredients. Moreover, defatted BSF larvae meal could be beneficial in FM replacement.

## Figures and Tables

**Table 1:** Formulation of experimental diets in feeding trial 1.

Ingredients	Control	Control+L	10PD	20PD	30PD
Fish meal (65% CP) ^1^	64.00	64.00	56.87	49.75	42.62
PDBM	-	-	10.00	20.00	30.00
Fish oil	8.00	8.00	8.76	9.51	10.27
Palm kernel oil	-	4.35	2.90	1.45	-
Corn oil	4.35	-	-	-	-
Starch	13.15	13.15	10.97	8.79	6.61
Taurine	1.00	1.00	1.00	1.00	1.00
Vitamin mix ^2^	0.80	0.80	0.80	0.80	0.80
Mineral mix ^3^	0.40	0.40	0.40	0.40	0.40
Choline chloride	0.10	0.10	0.10	0.10	0.10
Vitamin C derivatives	0.10	0.10	0.10	0.10	0.10
NaH_2_PO_4_	0.80	0.80	0.80	0.80	0.80
KH_2_PO_4_	0.80	0.80	0.80	0.80	0.80
Calcium lactate	1.50	1.50	1.50	1.50	1.50
CMC ^4^	5.00	5.00	5.00	5.00	5.00
Total	100.00	100.00	100.00	100.00	100.00
Proximate composition (%)					
Moisture	3.3	3.2	4.0	3.7	4.8
Crude protein	46.7	46.5	46.2	46.1	45.1
Crude fat	18.8	17.6	17.4	17.5	20.2
Ash	11.1	11.2	11.1	11.0	11.0
NFE + crude fiber ^5^	20.1	21.4	21.3	21.7	19.0
Fatty acid composition (% on a dried basis)					
C12:0	0.61	2.68	2.74	2.79	2.85
C16:0	2.85	2.73	2.95	3.18	3.40
C18:1	4.21	3.60	4.39	5.18	5.97
C22:6	2.35	2.35	2.35	2.35	2.35

^1^ Peruvian fish meal provided by Shintoa Corporation, Tokyo, Japan. ^2^ Vitamin mix (per 1000 mg of mix): thiamin·HCl, 8.0 mg; riboflavin, 20.0 mg; pyridoxine·HCl, 10.0 mg; nicotinic acid, 80.0 mg; calcium d-pantothenate, 40.0 mg; biotin (2%), 40.0 mg; folic acid, 1.5 mg; cyanocobalamin (2%), 12.5 mg; sodium calcium ascorbyl-2-phosphate, 200.0 mg; inositol, 200.0 mg; retinol powder (1,000,000 IU/g), 5.0 mg; cholecalciferol powder (500,000 IU/g), 1.0 mg; tocopherol (50%), 150.0 mg; menadion, 4.0 mg; cellulose, 228.0 mg (SINRA, Fukuoka, Japan). ^3^ Mineral mix (per 100 g of mix): NaCl, 5.0 g; MgSO_4_, 74.5 g; FeC_6_H_5_O7·_n_H_2_O, 7.5 g; C4H2FeO4, 5.0 g; ZnSO_4_·7H_2_O, 1765 mg; MnSO_4_·5H_2_O, 810 mg; CuSO_4_·5H_2_O, 155 mg; AlCl_2_·6H_2_O, 50 mg; CoCl_2_·6H_2_O, 5 mg; KlO_3_, 15 mg; (CH_3_CHOCOO)_2_Ca·5H_2_O, 2200 mg; Cellulose, 3.0 g (SINRA, Fukuoka, Japan). ^4^ Carboxymethyl-cellulose as a pelleting agent. ^5^ Nitrogen free extract (NFE) + crude fiber = 100 − (crude protein + crude fat + ash). Values for proximate composition were means of duplicate analyses. Values for fatty acid composition were calculated using the manufacture’s values. Abbreviations: PDBM, partially defatted black soldier fly larvae meal.

**Table 2:** Formulation of experimental diets in feeding trial 2.

Ingredients	Control	Control+L	10PD	20PD	10CD	20CD
Fish meal (65% CP) ^1^	64.00	64.00	57.18	50.36	57.18	50.36
PDBM	-	-	10.00	20.00	-	-
CDBM	-	-	-	-	8.02	16.03
Fish oil	8.00	8.00	8.74	9.49	8.74	9.49
Palm kernel oil	-	4.37	2.19	-	3.74	3.11
Corn oil	4.37	-	-	-	-	-
Starch	9.00	9.00	9.00	9.00	9.00	9.00
Cellulose	4.13	4.13	2.39	0.65	2.82	1.51
Taurine	1.00	1.00	1.00	1.00	1.00	1.00
Vitamin mix ^2^	0.80	0.80	0.80	0.80	0.80	0.80
Mineral mix ^3^	0.40	0.40	0.40	0.40	0.40	0.40
Choline chloride	0.10	0.10	0.10	0.10	0.10	0.10
Vitamin C derivatives	0.10	0.10	0.10	0.10	0.10	0.10
NaH_2_PO_4_	0.80	0.80	0.80	0.80	0.80	0.80
KH_2_PO_4_	0.80	0.80	0.80	0.80	0.80	0.80
Calcium lactate	1.50	1.50	1.50	1.50	1.50	1.50
CMC ^4^	5.00	5.00	5.00	5.00	5.00	5.00
Total	100.00	100.00	100.00	100.00	100.00	100.00
Proximate composition (%)						
Moisture	4.3	4.6	4.0	4.0	3.8	4.2
Crude protein	44.8	44.7	46.5	46.1	45.9	44.4
Crude fat	19.4	19.1	19.8	19.7	19.1	18.6
Ash	2.1	2.0	2.2	2.1	2.0	1.9
NFE + crude fiber ^5^	29.5	29.6	27.6	28.1	29.3	30.9
Fatty acid composition (% on a dried basis)						
C12:0	0.61	2.69	2.40	2.10	2.61	2.52
C16:0	2.29	0.39	0.37	0.34	0.98	1.58
C18:1	5.05	4.99	4.98	4.98	5.06	5.14
C22:6	2.35	2.35	2.35	2.35	2.35	2.35

^1^ Peruvian fish meal provided by Shintoa Corporation, Tokyo, Japan. ^2^ Vitamin mix (per 1000 mg of mix): thiamin·HCl, 8.0 mg; riboflavin, 20.0 mg; pyridoxine·HCl, 10.0 mg; nicotinic acid, 80.0 mg; calcium d-pantothenate, 40.0 mg; biotin (2%), 40.0 mg; folic acid, 1.5 mg; cyanocobalamin (2%), 12.5 mg; sodium calcium ascorbyl-2-phosphate, 200.0 mg; inositol, 200.0 mg; retinol powder (1,000,000 IU/g), 5.0 mg; cholecalciferol powder (500,000 IU/g), 1.0 mg; tocopherol (50%), 150.0 mg; menadion, 4.0 mg; cellulose, 228.0 mg (SINRA, Fukuoka, Japan). ^3^ Mineral mix (per 100 g of mix): NaCl, 5.0 g; MgSO_4_, 74.5 g; FeC_6_H_5_O7·_n_H_2_O, 7.5 g; C4H2FeO4, 5.0 g; ZnSO_4_·7H_2_O, 1765 mg; MnSO_4_·5H_2_O, 810 mg; CuSO_4_·5H_2_O, 155 mg; AlCl_2_·6H_2_O, 50 mg; CoCl_2_·6H_2_O, 5 mg; KlO_3_, 15 mg; (CH_3_CHOCOO)_2_Ca·5H_2_O, 2200 mg; Cellulose, 3.0 g (SINRA, Fukuoka, Japan). ^4^ Carboxymethyl-cellulose as a pelleting agent. ^5^ Nitrogen free extract (NFE) + crude fiber = 100 − (crude protein + crude fat + ash). Values for proximate composition were means of duplicate analyses. Values for fatty acid composition were calculated using the manufacture’s values. Abbreviations: PDBM, partially defatted black soldier fly larvae meal; CDBM, completely defatted black soldier fly larvae meal.

**Table 3:** Proximate composition of black soldier fly larvae meal in this study.

Proximate Composition (% on a Dried Basis)	PDBM	CDBM	FM
Crude protein	49.0	60.6	72.5
Crude fat	23.2	8.3	8.6
Ash	1.8	2.1	18.5

Values for proximate composition were means of duplicate analysis. Abbreviations: PDBM, partially defatted black soldier fly larvae meal; CDBM, completely defatted black soldier fly larvae meal; FM, fish meal.

**Table 4:** Amino acid (AA) profile of black soldier fly larvae meal in this study.

Components (% of Total AAs)	PDBM	FM
Ala	8.6	7.0
Arg	4.7	6.5
Asp	9.1	9.5
Cys	1.0	1.0
Glu	13.0	13.2
Gly	6.0	7.7
His	2.7	3.3
Ile	4.6	4.3
Leu	7.4	8.2
Lys	6.5	8.3
Met	1.8	3.1
Phe	4.4	4.3
Pro	6.4	5.0
Ser	4.4	4.3
Thr	4.2	4.6
Trp	1.6	1.3
Tyr	6.8	3.3
Val	6.7	5.2

Abbreviations: PDBM, partially defatted black soldier fly larvae meal; FM, fish meal.

**Table 5:** Fatty acid (FA) profile of black soldier fly larvae meal in this study.

Components (% of Total FAs)	Oil from PDBM	Fish Oil
Saturated fatty acid		
10:0	0.8	-
12:0	29.2	4.1
14:0	7.6	-
15:0	0.1	0.5
16:0	14.0	15.6
17:0	0.1	0.7
18:0	2.7	3.6
20:0	0.2	0.3
22:0	-	-
Total	54.7	24.8
Monounsaturated fatty acid		
14:1	0.1	-
16:1	1.2	5.0
17:1	0.1	0.5
18:1	40.2	19.4
20:1	-	5.2
22:1	-	5.0
24:1	-	0.6
Total	41.5	35.7
Polyunsaturated fatty acid		
ω-3 fatty acid		
16:3n-3	0.1	-
18:3n-3	3.5	1.0
20:3n-3	-	0.2
20:4n-3	-	0.7
20:5n-3	-	7.5
21:5n-3	-	0.3
22:5n-3	-	1.9
22:6n-3	-	15.7
Total	3.6	27.3
ω-6 fatty acid		
18:2n-6	-	2.8
20:2n-6	-	0.3
20:3n-6	-	0.2
20:4n-6	-	1.1
22:5n-6	-	0.6
Total	-	5.0
Others		
16:2	-	0.3
16:3	-	0.2
16:4	-	0.3
Total	-	0.8
Not identified	0.2	4.3

“-” indicates lower limit of quantitation: 1 mg/g. Abbreviation: PDBM, partially defatted black soldier fly larvae meal.

**Table 6:** Growth of juvenile yellowtail in feeding trial 1.

		Control	Control+L	10PD	20PD	30PD
*N*	Initial	60	60	60	60	60
	3 wks	60	60	59	60	60
	6 wks	60	58	57	43	57
	12 wks	59	58	54	41	56
FL (cm)	Initial	6.6 ± 0.3 ^a^	6.6 ± 0.4 ^a^	6.6 ± 0.3 ^a^	6.5 ± 0.3 ^a^	6.6 ± 0.3 ^a^
	3 wks	10.0 ± 0.5 ^a^	9.2 ± 0.6 ^c^	9.5 ± 0.6 ^b^	8.8 ± 0.7 ^d^	8.3 ± 0.7 ^e^
	6 wks	15.2 ± 1.1 ^a^	13.8 ± 1.0 ^a^	13.4 ± 1.0 ^a^	12.5 ± 1.0 ^b^	11.6 ± 1.1 ^c^
	12 wks	21.2 ± 2.1 ^a^	20.4 ± 1.5 ^b^	19.2 ± 1.9 ^c^	17.8 ± 1.8 ^d^	16.6 ± 1.7 ^e^
BW (g)	Initial	2.9 ± 0.5 ^a^	2.9 ± 0.5 ^a^	3.0 ± 0.5 ^a^	2.8 ± 0.4 ^a^	3.1 ± 0.5 ^a^
	3 wks	11.8 ± 2.2 ^a^	9.0 ± 2.2 ^c^	10.0 ± 2.0 ^b^	7.7 ± 1.8 ^d^	6.4 ± 1.7 ^e^
	6 wks	47.5 ± 10.3 ^a^	34.1 ± 8.2 ^a^	31.7 ± 7.7 ^a^	24.6 ± 6.5 ^b^	19.8 ± 6.0 ^c^
	12 wks	132.8 ± 41.2 ^a^	116.9 ± 27.4 ^b^	98.3 ± 31.2 ^c^	73.9 ± 25.3 ^d^	59.0 ± 20.9 ^e^
Specific growth rate (% day^−1^)		4.39	4.24	4.03	3.75	3.40
Feed conversion ratio		0.99	0.96	1.00	1.23	1.14

Values for FL and BW are represented by means ± S.D. Different letters in row indicate statistically significant differences according to Kruskal–Wallis test and Mann–Whitney test with Holm’s correction for post hoc comparison (*p* < 0.05). Abbreviations: FL, fork length; BW, body weight.

**Table 7:** Growth of juvenile yellowtail in feeding trial 2.

		Control	Control+L	10PD	20PD	10CD	20CD
*N*	Initial	60	60	60	60	60	60
	2.5 wks	57	60	58	58	60	59
	5 wks	52	59	58	57	59	59
FL (cm)	Initial	7.6 ± 0.4 ^a^	7.7 ± 0.5 ^a^	7.8 ± 0.5 ^a^	7.7 ± 0.5 ^a^	7.8 ± 0.5 ^a^	7.8 ± 0.5 ^a^
	2.5 wks	11.4 ± 0.7 ^a, b^	11.6 ± 0.8 ^a^	11.2 ± 0.8 ^a, b^	10.7 ± 0.8 ^c^	11.1 ± 0.8 ^b, c^	11.2 ± 0.7 ^b^
	5 wks	15.6 ± 0.9 ^a^	15.7 ± 0.9 ^a^	14.8 ± 1.1 ^b^	13.8 ± 1.1 ^c^	14.8 ± 1.0 ^b^	14.6 ± 0.9 ^b^
BW (g)	Initial	5.0 ± 1.0 ^a^	5.4 ± 1.0 ^a^	5.4 ± 1.2 ^a^	5.2 ± 1.2 ^a^	5.4 ± 1.2 ^a^	5.4 ± 1.2 ^a^
	2.5 wks	18.2 ± 3.5 ^a, b^	18.7 ± 4.1 ^a^	16.8 ± 3.9 ^a, b^	14.6 ± 3.7 ^c^	16.7 ± 3.5 ^b, c^	17.0 ± 3.7 ^a,b^
	5 wks	50.8 ± 9.8 ^a^	49.6 ± 10.1 ^a^	41.8 ± 10.6 ^b^	32.9 ± 8.3 ^c^	41.9 ± 9.8 ^b^	39.6 ± 8.8 ^b^
Specific growth rate (% day^−1^)		6.61	6.32	5.85	5.25	5.85	5.69
Feed conversion ratio		1.06	1.15	1.07	0.97	1.10	1.09

Values for FL and BW are represented by means ± S.D. Different letters in row indicate statistically significant differences according to Kruskal–Wallis test and Mann–Whitney test with Holm’s correction for post hoc comparison (*p* < 0.05). Abbreviations: FL, fork length; BW, body weight.

## Data Availability

The data that support the findings of this study are available from the corresponding author, upon reasonable request.
